# Environmental Pollution: An Under-recognized Threat to Children’s Health, Especially in Low- and Middle-Income Countries

**DOI:** 10.1289/ehp.1510517

**Published:** 2016-03-01

**Authors:** William A. Suk, Hamid Ahanchian, Kwadwo Ansong Asante, David O. Carpenter, Fernando Diaz-Barriga, Eun-Hee Ha, Xia Huo, Malcolm King, Mathuros Ruchirawat, Emerson R. da Silva, Leith Sly, Peter D. Sly, Renato T. Stein, Martin van den Berg, Heather Zar, Philip J. Landrigan

**Affiliations:** 1 Hazardous Substances Research Branch, Superfund Research Program, National Institute for Environmental Health Sciences, National Institutes of Health, Department of Health and Human Services, Research Triangle Park, USA; 2 Department of Allergy and Clinical Immunology, Ghaem Hospital, Mashhad University of Medical Sciences, Mashhad, Iran; 3 Water Research Institute, Council for Scientific and Industrial Research, Accra, Ghana; 4 Institute for Health and the Environment, University at Albany, Rensselaer, New York, USA; 5 Universidad Autónoma de San Luis Potosí, San Luis Potosí, Mexico; 6 Department of Preventive Medicine, Ewha Womans University School of Medicine, Seoul, Republic of Korea; 7 Shantou University Medical College, Shantou, China; 8 Faculty of Health Sciences, Simon Fraser University, Burnaby, British Columbia, Canada; 9 Chulabhorn Research Institute, Bangkok, Thailand; 10 Department of Pediatrics, School of Medicine, University of Caxias do Sul, Caxias do Sul, Brazil; 11 Children’s Health and Environment Program, Queensland Children’s Medical Research Institute, University of Queensland, Brisbane, Australia; 12 Centro Infant, Biomedical Research Institute, Pontifícia Universidade Católica do Rio Grande do Sul, Porto Alegre, Brazil; 13 Institute for Risk Assessment Sciences, Utrecht University, Utrecht, Netherlands; 14 Department of Paediatrics and Child Health, Red Cross War Memorial Children’s Hospital, MRC Unit on Child and Adolescent Health, University of Cape Town, Cape Town, Republic of South Africa; 15 Arnhold Global Health Institute, Icahn School of Medicine at Mount Sinai, New York, New York, USA.

## Abstract

Exposures to environmental pollutants during windows of developmental vulnerability in early life can cause disease and death in infancy and childhood as well as chronic, non-communicable diseases that may manifest at any point across the life span. Patterns of pollution and pollution-related disease change as countries move through economic development. Environmental pollution is now recognized as a major cause of morbidity and mortality in low- and middle-income countries (LMICs). According to the World Health Organization, pollution is responsible for 8.9 million deaths around the world each year; of these, 94% (8.4 million) are in LMICs. Toxic chemical pollution is growing into a major threat to children’s health in LMICs. The disease and disability caused by environmental pollution have great economic costs, and these costs can undercut trajectories of national development. To combat pollution, improved programs of public health and environmental protection are needed in countries at every level of development. Pollution control strategies and technologies that have been developed in high-income countries must now be transferred to LMICs to assist these emerging economies to avoid the mistakes of the past. A new international clearinghouse is needed to define and track the health effects of pollution, quantify the economic costs of these effects, and direct much needed attention to environmental pollution as a risk factor for disease.

## Introduction

The worst industrial accident in the world occurred in 1984 in a pesticide plant in Bhopal, India, where 200,000 people were exposed to methyl isocyanate. The gas leak and explosion at the plant caused more than 6,000 deaths, and another 50,000 people suffered long-term health effects (Dhara et al. 2002).

Exposures to ambient air pollution, toxic chemicals, and pesticides generally have been problems that cause disease in high-income countries (HICs). Was the Bhopal disaster an anomaly? Or was it an early indicator of an emerging global pattern in which environmental pollution and toxic chemicals are becoming a greater source of health risk in low- and middle-income countries (LMICs)?

To explore these questions, the authors considered data on patterns of environmental exposure and disease in 12 countries that are at different levels of development (United Nations Development Programme 2015): Australia, Brazil, Canada, China, Ghana, Iran, Mexico, South Africa, South Korea, Switzerland, Thailand, and the United States. These data were collected from official sources in each country, from data compiled through the Global Burden of Disease (GBD) project (Lim et al. 2012; Lozano et al. 2012; Murray et al. 2012, 2015), and from World Health Organization sources (WHO 2005, 2009, 2014a, 2014b, 2014c, 2014d) that include physicians and scientists who are members of the WHO Collaborating Centres for Children’s Environmental Health (CEH; http://www.niehs.nih.gov/research/programs/geh/partnerships/network/index.cfm). The WHO has established these centers over the past decade in countries at all levels of industrial and economic development. The WHO Collaborating Centres (WHO CCs) are now forming into a network to ensure effective collaboration and coordination of research efforts (Sly et al. 2014). To date, the CEH WHO CCs are in Australia, Japan, Mexico, Republic of Korea, Thailand, United States, and Uruguay. A proposed WHO CC in Brazil is currently in the application process. The network is formally coordinated by the WHO CC at the National Institute of Environmental Health Sciences (http://www.niehs.nih.gov/research/programs/geh/partnerships/).

## Discussion

Patterns of environmental pollution and of the diseases caused by pollution vary greatly from country to country. National income and level of development are critical factors responsible for these sharp differences (Barreto 2004; De Maio 2011).

### High-Income Countries

The principal pediatric diseases seen today in HICs are chronic, non-communicable diseases (NCDs) (WHO 2009). These diseases include birth defects—a leading cause of infant death in the United States (CDC 2006)—and asthma, which has been increasing in prevalence since 1980 (Akinbami 2002; Moorman et al. 2012). In addition, the reported prevalence of neurodevelopmental disorders, including dyslexia, mental retardation, attention deficit/hyperactivity disorder, and autism, have also increased sharply (CDC 2014), as have leukemia and brain cancer among children (Siegel et al. 2016). Childhood obesity more than doubled from 1980 to 2012 (CDC 2015), and its sequel, type 2 diabetes, has become epidemic (Hu et al. 2015; Beller 2000). The major diseases of adults in these countries are also NCDs—heart disease, stroke, cancer, diabetes, and chronic lung disease (Kelly et al. 2012).

### Low- and Middle-Income Countries

In contrast, indoor air pollution and contaminated drinking water have been the major environmental risk factors for disease in LMICs, as clearly demonstrated in recent analyses in Ghana and South Africa (WHO 2005, 2014a, 2014d). Malnutrition and the high incidence and prevalence of parasitic and vector-borne diseases have also been major threats to human health (Pronczuk et al. 2011; WHO 2009, 2014a): These health problems are still major contributors to the burden of disease in LMICs.

### Toxic Chemicals and Pesticides

Toxic chemicals and pesticides have long been important environmental pollutants in HICs, and thousands of these substances have been disseminated widely into the environment over the past century (Fischetti 2010). This long-standing concentration of toxic synthetic chemicals in the environment in HICs reflects the geographic origins of the chemical manufacturing industry, which began in Western Europe in the late 19th and early 20th centuries and then spread in the 20th century to North America, Japan, and Australia (Aftalion 2001). Many of the synthetic chemicals in widest use in these countries have never undergone any safety testing, and their potential toxicity is not known (Landrigan and Goldman 2011). Only about 20% have been screened for developmental toxicity (Goldman 1998). Scientists know even less about the possible synergistic effects of simultaneous exposures to multiple untested synthetic chemicals. National biomonitoring surveys conducted in the United States have documented several hundred synthetic chemicals in detectable quantities in the bodies of virtually all Americans of all ages (CDC 2009).

Toxic chemicals have been linked to numerous diseases through toxicological and epidemiological studies, and the list is growing as research into environmental causes of non-communicable disease continues. The likelihood is high that there are additional diseases and disabilities caused by widely used synthetic chemicals where the etiologic associations have not yet been recognized (Grandjean and Landrigan 2014). Chemicals of particular concern include organic chemicals that persist in the environment long after their production and use have been stopped, such as polychlorinated biphenyls, and non-persistent chemicals to which individuals are constantly exposed, such as the plastic components and plasticizers bisphenol A, other bisphenols, and phthalates. Exposures to these organic chemicals have been associated with increased risk of diabetes (Lee et al. 2007, 2012, 2014), hypertension (Goncharov et al. 2011), cardiovascular disease (Lang et al. 2008; Lind et al. 2012), obesity (Newbold 2010; Trasande et al. 2012), and cancer (Lauby-Secretan et al. 2013).

### Global Spread of Toxic Chemicals and Pesticides

In the past decade, with the globalization of trade, the spread of the Western life style, and the increasing globalization of the chemical manufacturing industry, toxic chemicals, highly hazardous pesticides, and chemical wastes, which previously were found only in developed countries, have been infiltrating LMICs with increasing rapidity (Spitz 2003). The manufacture and use of chemicals are shifting to LMICs, where labor costs are low and environmental and public health protections are few (Cole and Eliott 2005; Cole et al. 2010; Cole 2004; Kearsley and Riddel 2010). Chemical and pesticide pollution are increasing in LMICs, and hazardous wastes, including electronic waste, are accumulating (Grant 2014; Heacock et al. 2015; Luzardo et al. 2014; Perkins et al. 2014). At the same time, pollution-related chronic diseases such as asthma, heart disease, stroke, and cancer are becoming epidemic in countries where they were previously seldom seen (De Maio 2011; Kelly et al. 2012; Landrigan and Fuller 2014; Lim et al. 2012; Murray et al. 2015). The once separate patterns of disease in LMICs and HICs are converging (Dhara et al. 2002).

Tragic episodes of occupational and environmental exposure to toxic chemicals have resulted from the movement of the chemical industry to LMICs and have caused great damage. These include the devastating events such as the Bhopal disaster in India, and chronic, slowly unfolding tragedies such as the exposure of more than 1 million people to chrysotile asbestos in China, South and Southeast Asia, and sub-Saharan Africa (Frank and Joshi 2014). The new reality in global health is that NCDs are becoming major health problems in all countries around the world (Lim et al. 2012; Murray et al. 2015). In LMICs, especially those that are undergoing rapid industrialization, high risk of NCDs results in a double burden of disease, adding new threats to such age-old problems as infectious disease, inadequate clean drinking water, and poor nutrition (Pronczuk et al. 2011; Suk et al. 2003).

### Toxic Chemicals and Children’s Health in LMICs

A shift toward pollution-related diseases in developing countries presents a special problem for children who are already vulnerable because of inadequate nutrition and lack of access to clean drinking water. For children, exposures to environmental pollutants can be especially dangerous, as they are more sensitive than are adults to pesticides and other chemicals (reviewed in Landrigan and Goldman 2011). They intake more food, air, and water per pound than adults, and thus have greater exposures to toxic chemicals for their body weight. In addition, their metabolic pathways are immature, their early developmental processes are easily disrupted, and they have more time to develop chronic diseases than do adults (Landrigan and Goldman 2011). The concept that adverse environmental exposures in early life increase risk for disease outcomes in later life is not new and has been formalized in the developmental origins of health and disease (DOHAD) concept (Barker 2004). There are now many well-studied examples of DOHAD in the literature, including the increased risk of obesity and cardiovascular disease in adult life that were the consequence of maternal malnutrition during pregnancy during the Dutch Famine. Cancers and non-cancerous lung diseases also have been shown to result from prenatal exposure to high concentrations of arsenic (reviewed in Boekelheide et al. 2012).

Such toxic environmental exposures before birth or in early postnatal life can cause short-term death from acute disease in infancy and childhood as well as chronic NCDs that can manifest at any point across the human lifespan (Boekelheide et al. 2012; Grandjean and Landrigan 2014). Indeed, low-dose exposure occurring during developmental windows of susceptibility—brief, precisely timed periods in embryonic, fetal, and early postnatal life when vital organs are sculpted through highly choreographed and tightly scheduled developmental processes—have been shown to have far greater effects on health than high-dose exposures to the same chemicals among adults (Ho et al. 2012). Major acute diseases associated with environmental pollution in early life include pneumonia (Darrow et al. 2014; Fuertes et al. 2014) and diarrheal disease (Lanata et al. 2013). Chronic NCDs associated with environmental exposures in early life include neurobehavioral disorders (Bouchard et al. 2011; Braun et al. 2009; Engel et al. 2010; Jacobson and Jacobson 1996; Rauh et al. 2011; Grandjean and Landrigan 2014), adult and pediatric asthma (Gauderman et al. 2004), hypertension, obesity, diabetes, cardiovascular disease (Barker 2004), and cancer (Smith et al. 2012).

### Global Climate Change and Health

Global climate change could further exacerbate health risks from toxic environmental exposures, especially in LMICs, by increasing concentrations of many chemicals in water, air, and sediment (Noyes et al. 2009), as well as by imposing additional stress to individuals’ immune, endocrine, and neurological systems that may leave some even more sensitive to the pollutants they encounter (Hooper et al. 2013).

### Cost of Pollution

The diseases caused by pollution impose great economic costs on countries around the world—direct medical costs, opportunity costs reflecting the diminished productivity of populations damaged by pollution, and costs to health care systems (Jacobson and Jacobson 1996; Engel et al. 2010; Rauh et al. 2011; Bouchard et al. 2011; Trasande and Liu 2011; Landrigan and Fuller 2014).

The widespread pollution in a number of rapidly industrializing LMICs can result in adverse health effects, including damage to the brains, lungs, and other organ systems, for large numbers of persons (Laborde et al. 2015; Lanphear et al. 2000). This damage can result in diminished economic productivity of entire countries (Grosse et al. 2002; Landrigan and Fuller 2014; Trasande and Liu 2011; WHO 2010). For example, it is estimated that persons exposed to lead in countries that used leaded gasoline suffered widespread low-grade lead poisoning that resulted in cognitive impairment and reduced the population mean IQ in those countries by about five points (Lanphear et al. 2000; WHO 2010). A downward shift in cognitive function of this magnitude across an entire population has the effect of reducing, by more than 50%, the number of persons with IQ scores above 130, while at the same time increasing by more than 50% the number of persons with IQ scores below 70 (Weiss 1982). Such widespread cognitive impairment can reduce the intelligence and lifelong economic productivity of entire generations, thus undermining the developmental trajectory of whole societies (Landrigan and Fuller 2014).

Worldwide, environmental pollution is insufficiently appreciated and inadequately quantified as a cause of disease. It is estimated that pollution (the joint effects of household air pollution and ambient air pollution) is responsible for 7 million deaths per year (WHO 2014a, 2014d). This total is greater than the number of deaths due to HIV/AIDS, malaria, and tuberculosis combined (WHO 2014b, 2014c). Moreover, the overwhelming majority of the deaths attributed to ambient air pollution (88%) occurred in LMICs, and almost all of the deaths attributable to household air pollution were in LMICs (WHO 2014a).

### Need for a Global Pollution Control Strategy

Despite these sobering facts, pollution receives far less attention in national and international health and development assistance programs than do AIDS, malaria, and tuberculosis (Landrigan and Fuller 2014). Pollution, as a risk factor for health effects, is often undercounted because of the very high standard of proof required in the public policy arena to establish etiologic associations between pollution and illness and to bring about effective remedial action. Examples are provided by the protracted debates over whether lead in gasoline causes subclinical lead poisoning in children that preceded the removal of lead from gasoline (Needleman and Landrigan 1981), and the long-running controversy over whether particulate air pollution is responsible for cardiovascular disease and lung cancer (Hamra et al. 2014).

A second reason for the relative lack of attention paid to pollution as a cause of disease and death is that there is a lack of reliable exposure data at the population level, especially exposures in early life that may have occurred years or decades ago (Briggs 2003). Exposures to environmental toxicants can occur at low doses and are frequently ongoing from ingestion of food or water or from inhalation of pollutants in air. These factors make proving causation very difficult, especially when the adverse heath outcome from prenatal and early-life exposures occurred many years or decades later.

Yet another reason for the lack of attention paid to pollution is that the various components of pollution such as air pollution, water pollution, asbestos, and lead have been counted separately, one at a time (Landrigan and Fuller 2014). This disaggregated analytical approach is typical of environmental health research, and it is consistent with the structure of most public health and environmental protection agencies, which have separate bureaus for air, water, and solid waste. An unintended consequence of this fragmentation is that it minimizes the total impact of pollution and thus fails to give pollution the attention it deserves in planning and policy making.

## Conclusions

Patterns of disease are changing rapidly in LMICs. Pollution-related chronic diseases are becoming more common. This shift presents a particular problem for children, who are proportionately more heavily exposed than are adults to environmental pollutants and for whom these exposures are especially dangerous. Better quantification of environmental exposures and stepped-up efforts to understand how to prevent exposures that cause disease are needed in LMICs and around the globe.

To confront the global problem of disease caused by pollution, improved programs of public health monitoring and environmental protection are needed in countries at every level of economic development. Pollution control strategies and technologies that have been developed and successfully deployed in HICs need to be transferred to LMICs, and their implementation must be adequately funded. Pollution control strategies in HICs have succeeded by controlling exposures at the source. For instance, lead has been removed from gasoline (Grosse et al. 2002), asbestos use has been sharply curtailed and banned in some countries (Frank and Joshi 2014), and air and water pollution have been reduced. Highly toxic pesticides have been replaced. These actions have produced tangible benefits for human health and the environment. Such strategies can also succeed in LMICs, as evidenced by experience with reducing lead in gasoline, with resulting declines in children’s blood lead levels in countries such as the Philippines, India, and Pakistan (Suk et al. 2003). Such pollution control strategies can be highly cost effective and can lift the economies of entire nations. For example, the removal of lead from gasoline has resulted in much lower body lead burdens in children. Current evidence suggests that lowering lead exposure has a direct effect on reducing the incidence of impaired cognitive development and increasing economic productivity (Grosse et al. 2002). In the United States, removing lead from gasoline has returned approximately $200 billion to the U.S. economy each year since 1980 (Grosse et al. 2002).

Constructing strong public health and environmental protection programs will require several elements at the country level. Tracking systems to monitor environmental pollution, actual exposures, and disease provide an essential foundation for these systems. Training physicians and other health care providers to recognize and manage diseases caused by environmental pollution is a second essential need. Legally mandated safety testing of new chemicals before they are introduced to commercial markets and of existing chemicals is a third essential pillar of chemical control (Landrigan and Goldman 2011). Assessment of toxicity must be followed by governmental regulation, as voluntary controls appear to be of little value (Ashford and Caldart 2008).

The international development agenda must put a higher priority on pollution control, with a level of attention at least equal to that assigned to HIV/AIDS, malaria, and tuberculosis control (Landrigan et al. 2015). The lack of attention given to pollution in the program priorities of major international organizations is striking (Landrigan et al. 2015; Landrigan and Fuller 2014), especially given the substantial impact and favorable cost-benefit ratio of many pollution control programs such as the removal of lead from gasoline (Grosse et al. 2002), the installation of stack scrubbers on coal-fired power plants, and national bans on asbestos (Frank and Joshi 2014, Trasande and Liu 2011). Pollution protections are urgently needed in LMICs, where chemical production and use are rapidly increasing and environmental and occupational safeguards are too few. Current legal structures in many countries fail to adequately protect workers, children, and other vulnerable populations against environmental threats to health (Landrigan and Goldman 2011).

At the international level, an argument can be made for the formation of a new international clearinghouse focused on tracking the global movement of highly toxic pollutants and on defining the health effects of environmental pollution (Grandjean and Landrigan 2014). This new agency could be modeled on the International Agency for Research on Cancer and be placed in a position to:

Assess industrial chemicals and other forms of pollution for potential health effects using a precautionary approach that emphasizes prevention and does not require absolute proof of toxicity.
Facilitate and coordinate epidemiological and toxicological studies.
Lead the urgently needed global programs for pollution prevention.

In summary, the adverse health consequences of exposure to environmental toxicants constitute a large and rapidly growing global problem, yet they receive insufficient attention in the global health and international development agendas. It is time to focus the world’s attention on the great and growing global problem of environmental pollution. We must set stringent, but feasible numerical targets for pollution control.

Pollution deserves as much attention as infectious diseases. And, the global response to pollution deserves the same degree of rigor as has been applied to such infectious diseases as AIDS, tuberculosis, and malaria. Focus by the international community on environmental pollution can save the lives of millions, cost effectively and predictably. The need is great. The time is now.
